# Clinical Case Report on the Use of Rezafungin in *Pneumocystis jirovecii* Pneumonia in a Critically Ill Patient

**DOI:** 10.3390/microorganisms14030683

**Published:** 2026-03-18

**Authors:** Rosario Fernández-Fernández, Roberto García-Martínez, Waldo Sánchez-Yebra Fernández, Natalia Chueca-Porcuna, Manuel Colmenero-Ruiz

**Affiliations:** 1 Servicio de Medicina Intensiva, Hospital Universitario Clínico San Cecilio (HUCSC), 18009 Granada, Spain; robbiegar27@gmail.com (R.G.-M.);; 2 Servicio de Microbiología, Hospital Universitario Clínico San Cecilio (HUCSC), 18009 Granada, Spain; waldosanchezyf@gmail.com (W.S.-Y.F.); naisses@yahoo.es (N.C.-P.); 3 Instituto de Investigación Biosanitaria ibs.GRANADA, 18009 Granada, Spain; 4 Spanish Consortium for Research on Infectious Diseases (CIBERINFEC), Madrid, Spain; 5 Faculty of Medicine, Universidad de Granada (UGR), 18016 Granada, Spain

**Keywords:** *Pneumocystis jirovecii* pneumonia, rezafungin, echinocandin, immunocompromised, intensive care, β-D-glucan, compassionate use

## Abstract

*Pneumocystis jirovecii* pneumonia (PJP) is a life-threatening opportunistic infection predominantly affecting immunocompromised patients. Trimethoprim–sulfamethoxazole (TMP–SMX) remains the standard therapy but is often limited by severe toxicity. Rezafungin, a next-generation echinocandin with a long half-life, has shown preclinical activity against Pneumocystis spp., but its use in humans remains largely unexplored. We report the case of a 67-year-old man with inflammatory bowel disease who developed severe PJP complicated by acute respiratory failure, septic shock, and multiorgan dysfunction following corticosteroid and biologic therapy. Standard TMP–SMX therapy was contraindicated due to renal impairment and pancytopenia. The patient received rezafungin via a compassionate-use programme, with serial monitoring of serum and bronchoalveolar β-D-glucan levels. Despite a partial biomarker response, the patient ultimately progressed to refractory acute respiratory distress syndrome and multiorgan failure. This case provides preliminary human data suggesting that rezafungin may offer a potential therapeutic option for PJP when standard treatment is contraindicated or poorly tolerated. Close clinical and biomarker monitoring is essential. Further clinical and experimental studies are warranted to determine its efficacy, optimal dosing, and safety in critically ill immunocompromised patients.

## 1. Introduction

*Pneumocystis jirovecii* pneumonia (PJP) is a potentially life-threatening opportunistic fungal infection that predominantly affects immunocompromised individuals and often results in severe respiratory impairment [1]. Those at risk include patients with underlying conditions that impair immunity, such as haematological malignancies, HIV infection or solid organ transplantation, as well as individuals receiving prolonged corticosteroid therapy or biological immunosuppressive agents. Clinically, PJP often presents with fever, non-productive cough and progressive dyspnoea, and can rapidly evolve into acute respiratory failure requiring intensive care support [2].

Clinically significant PJP arises in the setting of congenital or acquired cellular immunodeficiencies. Impairment of T-lymphocyte-mediated immunity and macrophage dysfunction are central to its pathogenesis. CD4+ T-cell lymphopenia is a well- established risk factor for disease development [3,4,5,6]. In people living with HIV, the widespread availability of effective antiretroviral therapy has markedly reduced the incidence of PJP, and prophylaxis is therefore recommended for individuals with CD4+ cell counts below 200 cells/µL [4].

In non-HIV immunocompromised populations, prolonged corticosteroid therapy is the strongest and most consistent risk factor [5]. The risk of PJP is dose-dependent: high-dose prednisone (>30 mg/day) or moderate doses for more than four weeks substantially increase susceptibility [6,7]. Other clinical scenarios, such as lymphoproliferative disorders, autoimmune diseases, and immunosuppression related to advanced therapies, further contribute to vulnerability through combined humoral and cellular immune dysfunction [8].

Rezafungin is an investigational next-generation echinocandin for the treatment and prophylaxis of invasive fungal infections, including PJP. It acts by inhibiting β-(1,3)-D- glucan synthesis, which is a fundamental structural component of the fungal cell wall. A key molecular modification from its parent compound, anidulafungin, gives rezafungin enhanced chemical stability and a significantly longer half-life. This enables high early drug exposure and once-weekly dosing, providing advantages over the echinocandins that are currently approved [9,10].

Although trimethoprim–sulfamethoxazole (TMP–SMX) remains the primary agent for both prophylaxis and treatment of PJP, concerns regarding its frequent toxicity, including bone marrow suppression, hepatotoxicity, nephrotoxicity, and severe hypersensitivity reactions, can limit its use [11,12]. In an open-label randomised controlled study, up to 42% of patients receiving TMP-SMX for PJP prophylaxis discontinued therapy due to adverse events [13]. This poses a substantial challenge in critically ill patients, in whom drug-related complications may be exacerbated.

While rezafungin’s antifungal activity covers a broad spectrum of Candida and Aspergillus species, similar to currently approved echinocandins [14,15], it is notable for demonstrating excellent activity against Pneumocystis species comparable to TMP-SMX, as observed in biofilms and animal models [16,17,18]. Its prolonged half-life (approximately 130 h in humans) and favourable safety profile support a front-loaded weekly dosing regimen, which may improve adherence and reduce toxicity in real-world settings [19,20,21,22].

Although preclinical evidence strongly supports the use of rezafungin in prophylaxis, its use in the treatment of PJP in humans remains undocumented. Here, we present a case study of a critically ill patient with severe PJP who developed septic shock and multiple organ failure. Standard therapy was contraindicated, and the patient ultimately received rezafungin through a compassionate-use programme. In parallel, early real-world utilisation data are emerging through international observational initiatives such as the FungiScope registry [23].

## 2. Case Presentation

The patient was a 67-year-old man with no significant past medical history who presented to the Emergency Department with a one-month history of gastrointestinal symptoms. His symptoms included diarrhoea (five to seven loose stools daily, occasionally containing blood and mucus), lower abdominal pain, and unintentional weight loss of approximately 4 kg. He also reported intermittent fever. Initial laboratory tests revealed markedly elevated C-reactive protein levels (CRP 250 mg/L) and a prerenal acute kidney injury (AKIN I), alongside a normal leukocyte count and procalcitonin level. An abdominal CT scan revealed inflammatory changes at the hepatic flexure and descending colon, as well as segmental thickening of the sigmoid colon. This raised the possibility of inflammatory or infectious colitis, but an underlying neoplastic process could not be ruled out.

The patient was initially admitted to the Gastroenterology department with a diagnosis of colitis of uncertain aetiology. A colon biopsy revealed positive cytomegalovirus (CMV) immunohistochemistry but negative CMV PCR. Due to the absence of a clear infectious aetiology, corticosteroid therapy was initiated to treat suspected inflammatory bowel disease. However, this resulted in a poor clinical response, with persistent moderate activity and microabscesses observed on repeat biopsy. Subsequently, a biological treatment with infliximab was started, leading to partial clinical and analytical improvement, but no definitive resolution.

Subsequently, the patient developed hypotension and a rapid clinical deterioration, prompting his first admission to the Intensive Care Unit (ICU) for haemodynamic support. Given the positive cytomegalovirus (CMV) immunohistochemistry on colonic biopsy in an immunosuppressed host, CMV colitis/reactivation was considered in the differential diagnosis, and intravenous ganciclovir was initiated; foscarnet was added transiently while virological confirmation was pending. In parallel, due to the severity of illness with suspected sepsis in the context of severe colitis (raising concern for bacterial translocation and/or an intra-abdominal source), empirical broad-spectrum antibacterial therapy with meropenem and linezolid was commenced. A secondary hyperinflammatory syndrome was considered; however, a formal diagnosis of secondary haemophagocytic lymphohistiocytosis (sHLH) was not established. Foscarnet was later discontinued due to electrolyte disturbances and repeatedly negative CMV PCR results.

**Figure 1:**
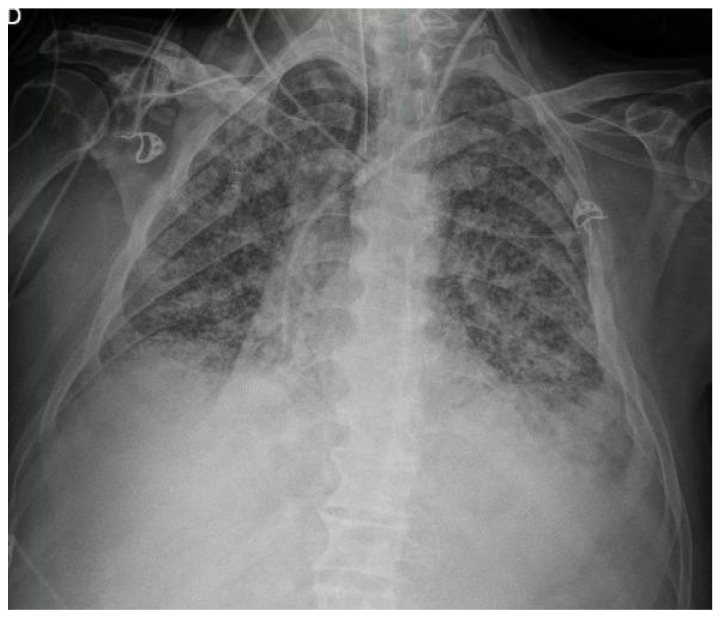
Chest radiograph obtained during the acute presentation, showing diffuse bilateral interstitial and alveolar infiltrates with a predominant perihilar distribution in an immunocompromised patient. Endovascular and airway devices are correctly positioned.

The patient’s clinical condition improved, and he was transferred back to the general ward. There, corticosteroid tapering continued and ustekinumab was initiated as a second-line biologic therapy. Shortly after receiving the infusion, the patient developed fever, cough, progressive dyspnoea, and severe hypoxaemia, which led to a second ICU admission for acute respiratory failure. Bedside lung ultrasound revealed a diffuse bilateral B-line pattern and a left basal C-pattern, which were consistent with alveolar involvement. Chest radiography confirmed bilateral infiltrates (Figure 1), and a presumptive diagnosis of drug-induced pneumonitis secondary to biological therapy was made.

The patient underwent bronchoalveolar lavage (BAL), and PJP was confirmed on BAL fluid by Gomori methenamine silver staining and PCR. Microbiological testing supported the suspicion of PJP in this immunocompromised patient by demonstrating markedly elevated serum beta-D-glucan levels (>600 pg/mL) with negative galactomannan. Fungal biomarker monitoring was included throughout the clinical course, with (1,3)-beta-D-glucan levels determined in both serum and BAL using the commercial colorimetric method Fungitell^®^ (Associates of Cape Cod, Falmouth, MA, USA). Empirical therapy was initiated along with trimethoprim–sulfamethoxazole (TMP-SMX) at PJP treatment doses and systemic corticosteroids.

Following the initial empirical treatment, the patient developed progressive renal dysfunction and pancytopenia, which were highly suggestive of TMP-SMX toxicity. Despite optimising ventilatory parameters, employing two cycles of prone positioning, and administering systemic corticosteroids for acute respiratory distress syndrome (ARDS), the lung remained non-compliant, resulting in persistent hypoxaemia and refractory respiratory failure.

Despite receiving optimal supportive care and non-invasive oxygen therapy, the patient’s oxygenation progressively worsened, necessitating endotracheal intubation and invasive mechanical ventilation 48 h after ICU admission. Even with protective ventilation and neuromuscular blockade, severe hypoxaemia persisted (PAO_2_/FiO_2_ approx 54 mmHg). Haemodynamic monitoring with pulse contour cardiac output (PiCCO) revealed a state of hyperdynamic shock with low systemic vascular resistance, necessitating vasoactive support and prone positioning, which only resulted in transient improvement.

In this challenging context, given the patient’s critical condition, the lack of a microbiological response to initial therapy, and the suspected toxicity of TMP-SMX, the institutional antimicrobial stewardship team (PROA-ICU) authorised compassionate use of rezafungin, a once-weekly echinocandin with a long half-life. The patient received a 400 mg dose of rezafungin after the experimental nature of the treatment and the available clinical data from the Phase III trial [24] were explained to the patient’s legal representative.

**Figure 2:**
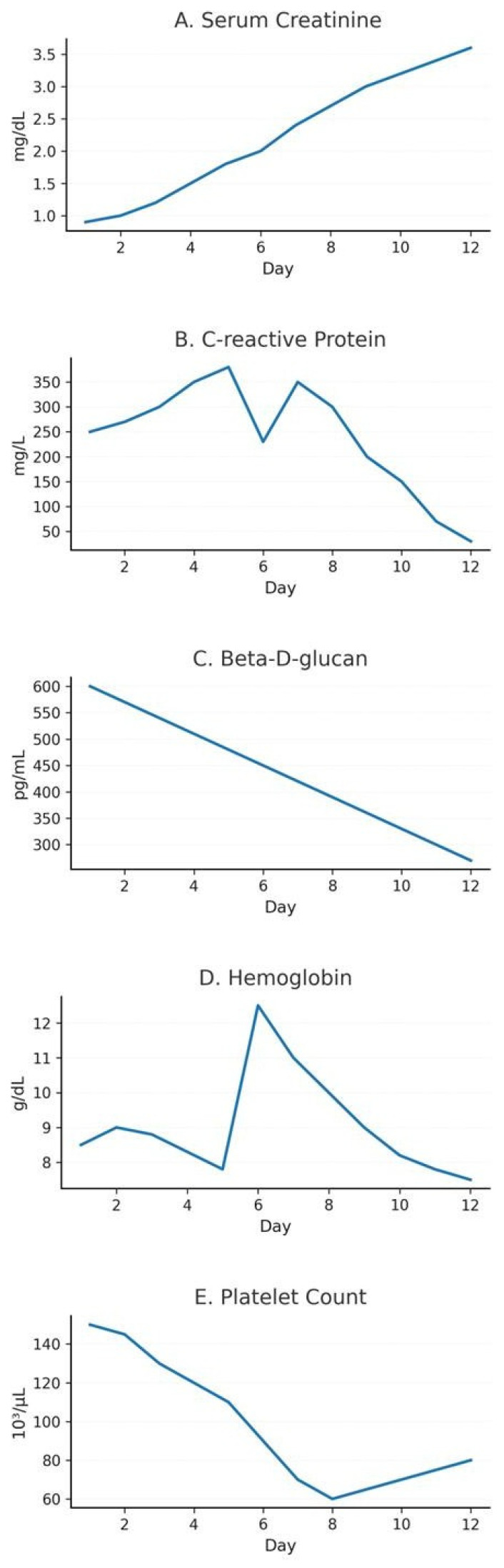
Temporal evolution of key laboratory parameters during ICU admission. (**A**) Serum creatinine levels showing progressive worsening consistent with acute kidney injury. (**B**) C-reactive protein concentrations display an initial inflammatory peak followed by a subsequent decline. (**C**) Serum β-D-glucan values were persistently elevated at presentation and showed a progressive decrease during antifungal therapy. (**D**) Haemoglobin variation throughout the clinical course, with a transient rise on Day 5 corresponding to the administration of blood products. (**E**) Platelet count showing an early downward trend, followed by partial recovery. Day 1 corresponds to the initiation of rezafungin.

Fungal biomarker monitoring was conducted throughout the clinical course: D-glucan levels were measured at diagnosis, during antifungal treatment, and during clinical follow-up in order to evaluate therapeutic response and correlation with clinical and radiological outcomes. The temporal evolution of key laboratory parameters during ICU admission is presented in Figure 2.

Despite therapeutic efforts, the patient’s clinical deterioration progressed and no significant haemodynamic improvement was demonstrated. The patient continued to exhibit severe gas exchange impairment and fibrotic changes consistent with late-phase acute respiratory distress syndrome (ARDS), concurrent with a flare of his inflammatory bowel disease that resulted in severe anaemia due to rectal bleeding.

Despite the introduction of rezafungin, the progression towards irreversible multi-organ failure led the multidisciplinary team and the patient’s relatives to limit further life- sustaining interventions. The patient subsequently passed away in the ICU due to refractory ARDS caused by a *Pneumocystis jirovecii* infection. Nevertheless, as clinical evidence in humans remains scarce and is limited to compassionate settings, this case provides an important initial observation on the potential usefulness of rezafungin in PJP when conventional alternatives are ineffective or contraindicated.

## 3. Discussion

*Pneumocystis jirovecii* pneumonia (PJP) remains a significant cause of morbidity and mortality in immunocompromised patients, including those with haematological malignancies, autoimmune or inflammatory diseases, and individuals undergoing prolonged immunosuppressive therapy. In this case, the patient had significant immunosuppression due to an underlying inflammatory bowel disease and recent exposure to corticosteroids, infliximab, and ustekinumab—conditions that greatly increase the risk of opportunistic fungal infections. The patient’s clinical presentation, characterised by acute respiratory failure, fever, and diffuse bilateral interstitial involvement, was highly consistent with severe PJP.

Trimethoprim–sulfamethoxazole (TMP–SMX) remains the gold standard for PJP treatment due to its well-established efficacy. However, its toxicity profile poses a significant clinical challenge, particularly in critically ill or multimorbid patients. Haematological, hepatic, and renal adverse effects frequently necessitate discontinuation or dose reduction, as occurred in this case, where the patient developed pancytopenia and progressive renal dysfunction shortly after treatment initiation. These complications ultimately limited the use of TMP–SMX at a time when optimal antifungal activity was essential [25,26].

During the first ICU admission, the patient’s haemodynamic deterioration occurred in the context of severe colitis and profound immunosuppression, raising concern for sepsis and possible CMV reactivation. In critically ill immunocompromised patients, sepsis-associated hyperinflammation can overlap clinically with secondary haemophagocytic lymphohistiocytosis (sHLH). Although sHLH was considered in this case, the available clinical data were insufficient to establish a formal diagnosis. Nonetheless, heightened clinical suspicion and early structured evaluation (including ferritin and other HLH-directed laboratory testing) may be warranted when systemic inflammation and cytopenias are disproportionate, as this may influence timely identification of triggers and adjunctive immunomodulatory strategies.

In this context, rezafungin—an investigational next-generation echinocandin with an exceptionally long half-life—has emerged as a promising treatment option. Although all echinocandins inhibit β-(1,3)-D-glucan synthesis, a vital component of fungal cell walls, rezafungin’s unique chemical properties provide it with enhanced metabolic stability and prolonged plasma exposure. These features support once-weekly dosing and could offer benefits to patients who are severely clinically frail or who struggle with daily multidrug regimens. Preclinical studies have shown rezafungin’s prophylactic and therapeutic activity against Pneumocystis species in murine models, achieving significant reductions in pulmonary fungal burden and preventing recurrence. Although these results have not yet been confirmed in human clinical trials, they indicate a possible role for rezafungin in situations where current treatments are contraindicated or poorly tolerated [27,28].

From a pharmacokinetic perspective, weekly administration of rezafungin sustains stable therapeutic concentrations in pulmonary tissue over prolonged durations, potentially enhancing patient adherence and minimising toxicity risks. Nevertheless, its definitive efficacy against *Pneumocystis jirovecii* has not yet been corroborated through large-scale studies. The unique composition of the cell wall in *Pneumocystis*, which significantly diverges from that of filamentous fungi, may lead to variability in the susceptibility of β- glucan targets to echinocandin therapy depending on the species and developmental stage. Consequently, the clinical application of preclinical findings should be approached with caution [24,29].

In this case, monitoring of fungal biomarkers revealed a dynamic correlation between clinical progression and levels of beta-D-glucan (BDG) in both serum and bronchoalveolar lavage (BAL) fluid. The (1→3)-β-D-glucan assay is a valuable quantitative biomarker of fungal burden, with diagnostic and prognostic utility in immunocompromised patients. Elevated BDG levels are a recognised feature of PJP, and serial measurements can assist in monitoring the response to antifungal therapy. Several studies have highlighted the usefulness of BDG in serum and BAL samples for diagnosis, prognostic stratification, and therapeutic assessment, particularly when integrated with molecular techniques and radiological evaluation. In parallel, C-reactive protein (CRP) decreased gradually during the second week after rezafungin initiation (Figure 2B), which may reflect partial attenuation of systemic inflammation; however, CRP is non-specific in critically ill patients and cannot be attributed to antifungal therapy alone. The progressive decline in BDG levels observed during rezafungin administration in this patient may indicate antifungal activity; however, the clinical progression towards irreversible respiratory failure ultimately overshadowed these findings [30,31,32,33].

This case underscores the urgent necessity to evaluate alternative antifungal therapies for patients experiencing severe toxicity or intolerance to TMP–SMX. In the ICU environment, drug–drug interactions, organ dysfunction, and the high incidence of multiorgan failure markedly limit the therapeutic options for conventional antifungals, thereby rendering innovative agents such as rezafungin valuable candidates for compassionate use programmes. Additionally, this case emphasises the critical importance of close interdisciplinary collaboration among intensivists, microbiologists, pharmacologists, and antimicrobial stewardship teams to ensure that decisions regarding novel or experimental therapies are ethically justified and grounded in evidence.

Although this case resulted in a fatal outcome due to refractory acute respiratory distress syndrome (ARDS) and multiorgan failure, it offers significant early clinical insights into the potential application of rezafungin in treating *Pneumocystis jirovecii* pneumonia (PJP) in critically ill, immunosuppressed patients. The biomarker response observed, together with preclinical evidence, indicates that rezafungin could serve as a therapeutic option when standard treatments are contraindicated or poorly tolerated. Nonetheless, the current lack of robust clinical evidence warrants that its use be confined to carefully monitored compassionate-use situations until further data becomes available.

## 4. Conclusions

This case study describes the compassionate use of rezafungin in an immunosuppressed patient with PJP who could not tolerate standard trimethoprim–sulfamethoxazole treatment. The positive clinical outcome observed after rezafungin was introduced suggests it could be beneficial in settings where conventional alternatives are not feasible or involve significant toxicity.

However, clinical evidence on the efficacy of rezafungin against *P. jirovecii* in humans is limited and is currently based on isolated case studies. This case highlights the need for more extensive clinical and experimental research to confirm its effectiveness in real- world settings, establish optimal dosing regimens and determine its long-term safety.

The management of PJP in immunocompromised patients remains a clinical challenge. The incorporation of new antifungals should be approached cautiously within compassionate use programmes and with close microbiological and pharmacological monitoring.

### Impact on Clinical and Intensive Care Units

In intensive care medicine, early identification and personalised management of opportunistic infections are crucial for improving prognosis. This case emphasises the importance of the key considerations summarised in Table 1.

This case exemplifies how pharmacological innovation can provide alternative solutions for complex patients, while also highlighting the need to rigorously document its true clinical impact before widespread adoption.

## Figures and Tables

**Table 1:** Key considerations for ICU practice when managing severe PJP with limited standard treatment options.

Domain	Practical Implication	Rationale (Case-Based)
Early recognition	Prompt recognition of PJP in non-HIV immunosuppressed patients	Rapid progression to severe hypoxemia/ARDS despite early therapy
Toxicity surveillance	Close monitoring for TMP–SMX toxicity (renal + haematologic)	Treatment-limiting AKI and pancytopenia required discontinuation
Alternative therapy pathway	Consider compassionate-use options when standard therapy is contraindicated	Limited alternatives in critical illness; multidisciplinary decision
Multidisciplinary stewardship	Engage ICU, microbiology, pharmacy, stewardship/ethics early	Supports documentation, dosing strategy, and risk–benefit assessment
Data generation	Contribute cases to registries/series	Evidence for rezafungin in human PJP remains scarce

## Data Availability

The data presented in this study are available on request from the corresponding author. The data are not publicly available due to privacy and ethical restrictions.
